# Screening kinase inhibitors identifies MELK as a prime target against influenza virus infections through inhibition of viral mRNA splicing

**DOI:** 10.3389/fmicb.2025.1600935

**Published:** 2025-06-05

**Authors:** Xuanye Yang, Xili Feng, Qianyun Liu, Lele An, Zhongren Ma, Xiaoxia Ma

**Affiliations:** ^1^Key Laboratory of Biotechnology and Bioengineering of State Ethnic Affairs Commission, Biomedical Research Center, Northwest Minzu University, Lanzhou, China; ^2^College of Life Science and Engineering, Northwest Minzu University, Lanzhou, China

**Keywords:** influenza virus, MELK, kinase inhibitor, antiviral agent, combination therapy

## Abstract

Influenza epidemics represent a significant threat to global public health, primarily caused by the influenza viruses A and B. Although antiviral drugs targeting the influenza virus, such as zanamivir and oseltamivir, are clinically available, the emergence of virus evolution and drug resistance necessitates the development of host-directed therapies. Protein kinases are essential components of host signaling pathways, including the orchestration of virus–host interactions. By screening a library of kinase inhibitors, we identified that OTS167, a pharmacological inhibitor of maternal embryonic leucine zipper kinase (MELK), strongly inhibits the infections caused by multiple influenza virus subtypes in cell culture. This antiviral activity was further confirmed by treatment with another MELK pharmacological inhibitor, MELK-8a, and siRNA-mediated MELK gene silencing. In mice challenged with the influenza A virus, treatment with OTS167 inhibited both viral replication and lung inflammation. Mechanistically, inhibition of MELK by OTS167 downregulates the downstream effector CDK1, thereby inhibiting influenza virus M1 mRNA splicing to reduce viral replication and virus particle assembly. Finally, we demonstrated that combining OTS167 with zanamivir or oseltamivir resulted in additive antiviral activity. In conclusion, we identified MELK as a crucial host kinase that supports the influenza virus infection. OTS167, a pharmacological inhibitor of MELK currently undergoing phase II clinical trials for treating cancer, potently inhibits influenza virus infections *in vitro* and in mice, representing a promising lead for developing novel influenza antivirals.

## 1 Introduction

Influenza viruses are classified into four types: A, B, C, and D. Influenza A virus (IAV) and influenza B virus (IBV) remain a substantial global health threat, causing widespread respiratory disease and contributing to significant burdens of morbidity and mortality in humans and animals (Liang, 2023). IBV primarily infects humans, whereas IAV is zoonotic. Concurrently, the global spread of the highly pathogenic avian influenza A H5N1 subtype in birds is considered a significant pandemic threat (Kupferschmidt, 2024). Alarmingly, H5N1 infections in more than 40 mammalian species have been reported, and it is causing widespread infections in dairy cows in the United States, with sporadic human cases identified (Garg et al., 2024; Jassem et al., 2024; Uyeki et al., 2024).

Influenza viruses are characterized by a single-stranded, segmented RNA genome consisting of eight segments that encode 10 viral proteins (Javanian et al., 2021). Viral neuraminidase is located on the surface of influenza viruses, which enables the virus to be released from the host cell by cleaving sialic acid groups. It is a prime viral target for therapeutic development, and several neuraminidase inhibitors, including oseltamivir and zanamivir, are clinically approved for treating influenza (Su et al., 2022). However, the evolution of influenza viruses mainly through antigenic shift and drift poses a significant challenge to the efficacy of these direct-acting antivirals due to the development of resistance (Liang, 2023). In parallel, the life cycle of influenza viruses heavily relies on cellular host factors. For example, the matrix protein 2 (M2) ion channel protein shares an RNA segment with matrix protein 1 (M1), and the IAV non-structural protein 1 (NS1) interacts with host proteins to utilize host splicing machinery, thereby regulating M1 mRNA splicing (Tsai et al., 2013; Dubois et al., 2014).

Significantly, many gene products from influenza viruses are extensively modified by host kinase-mediated phosphorylation. The reversible phosphorylation of specific serine, threonine, and tyrosine residues dynamically regulates viral proteins’ structure, function, and subcellular localization (Dey and Mondal, 2024). Previous studies have documented the crucial role of host protein kinases in the influenza virus life cycle, encompassing viral entry, genome replication, protein translation, and eventual viral budding (Li et al., 2019; Meineke et al., 2019; Dey and Mondal, 2024). The human kinome contains over 500 protein kinases, which play a pivotal role in regulating various cellular processes and pathophysiology (Sueca-Comes et al., 2022). Small-molecule kinase inhibitors have emerged as promising drug candidates for treating multiple diseases, particularly cancer (Roskoski, 2024).

This study aims to better understand how human kinases regulate influenza virus infection and identify host kinase-targeted antiviral therapeutics. We first screened a library of 172 kinase inhibitors in the human A549 lung cell line infected with the IAV PR/8 strain. We identified OTS167, a pharmacological inhibitor of maternal embryonic leucine zipper kinase (MELK), as one of the most potent inhibitors of IAV infection. OTS167 exerts broad-spectrum antiviral activity against both IAV and IBV strains. In mice challenged with IAV, OTS167 inhibited both viral replication and lung inflammation. We further demonstrated that inhibiting MELK by OTS167 downregulates CDK1 expression, ultimately disrupting the splicing of the viral M1 gene mRNA to inhibit viral replication and virus particle assembly. Finally, combining OTS167 with the clinically approved influenza drug oseltamivir or zanamivir exerted additive antiviral activity.

## 2 Materials and methods

### 2.1 Cells and viruses

Human lung adenocarcinoma A549 cells and Madin–Darby canine kidney (MDCK) cells were provided by the Biomedical Research Center, Northwest Minzu University, Lanzhou, China, and were maintained in Ham’s F12 nutrient medium (F12) and Dulbecco’s Modified Eagle’s Medium (DMEM) (Lanzhou Bailing Bio-Tech Company Limited, China) supplemented with 10% fetal bovine serum (FBS) (Lanzhou Minhai Bio-Engineering Co., Ltd., China). All cell lines were maintained at 37°C in a humidified 5% CO_2_ incubator using antibiotic-free culture medium.

Influenza virus A/Puerto Rico/8/1934 H1N1 (PR/8), A/Singapore/GP1908/2015 (IVR-180), and B/Washington/02/2019 were maintained by our laboratory and were propagated in 10-day-old embryonated chicken eggs at 37°C for 72 h.

### 2.2 Mice

Specific pathogen-free C57BL/6 mice were purchased from Lanzhou Veterinary Research Institute, Chinese Academy of Agricultural Sciences. Experiments involving mice were performed following the protocols and procedures reviewed and approved by the Animal Experiment Committee of the Laboratory Animal Centre, Lanzhou Veterinary Research Institute (Approval Number: LVRIAEC-2023-036).

### 2.3 Reagents and antibodies

All compounds were purchased from MedChemExpress (Shanghai, China). The MCE kinase inhibitor library is composed of 172 kinase inhibitors, with primary targets encompassing protein kinases (including VEGFR, EGFR, BTK, CDK, and Akt), lipid kinases (including PI3K, PI4K, and SK), and carbohydrate kinases (e.g., hexokinase) (Supplementary Table 1). Antibodies targeting IAV NP (GTX636247), HA (GTX127357), M1 (GTX125928), M2 (GTX125951), IBV NP, and HA (GTX128522) were purchased from GeneTex (Shanghai, China). The MELK antibody (ab108529) was purchased from Abcam (Shanghai, China). The CDK1 antibody (HY-P80611) and phospho-CDK1 (Tyr15) antibody (HY-P80796) were purchased from MedChemExpress (Shanghai, China). The β-tubulin antibody (A01030) was purchased from Abbkine (Wuhan, China). The GAPDH antibody (AP0066) was purchased from Bioworld (Nanjing, China). Horseradish Peroxidase (HRP) conjugated anti-rabbit IgG (A21020) and HRP conjugated anti-mouse IgG (A21010) were purchased from Abbkine (Wuhan, China).

### 2.4 Drug screening

A549 cells were plated in 12-well plates and cultured to near 80% confluence. The cells were subsequently infected with IAV PR/8 at a multiplicity of infection (MOI) of 0.1 in serum-free F12 medium for 2 h at 37°C. After removing the inoculum, cells were washed once with phosphate-buffered saline (PBS), and maintenance medium [F12 supplemented with 2 μg/mL L-(tosylamido-2-phenyl) ethyl chloromethyl ketone (TPCK)-treated trypsin] with the compound mixture was subsequently added. To minimize non-specific effects on host cells, we used a low concentration of 1 μM and treated for 36 h. Control treatments consisted of equal volumes of either ddH_2_O or dimethyl sulfoxide (DMSO) (Chen et al., 2020). Quantitative real-time PCR (RT-qPCR) was performed to measure IAV PR/8 genomic RNA levels.

### 2.5 RNA extraction, cDNA synthesis, and RT-qPCR

Total RNA was extracted using the Total RNA Extraction Kit (Solarbio, Beijing, China), and total RNA was then reverse transcribed into complementary DNA (cDNA) with the reverse transcription system from Vazyme (Nanjing, China). The RT-qPCR was performed with SYBR Green (Vazyme, Nanjing, China) following the instructions, and the reaction system was as follows: cDNA: 1 μL, forward primer (10 μM): 0.5 μL, reverse primer (10 μM): 0.5 μL, qPCR Master Mix: 10 μL, and RNase Free Water: 8 μL. RT-qPCR assays were performed, and the glyceraldehyde 3-phosphate dehydrogenase (GAPDH) gene was used as a reference gene (Eisenberg and Levanon, 2013; Hui et al., 2022). Relative gene expression was normalized to GAPDH using the formula 2^−∆∆CT^ (Livak and Schmittgen, 2001). All primers used in this study were validated via standard curve analysis, with their specific sequences detailed in Supplementary Table 2.

### 2.6 Western blot assay

Cells were harvested and lysed on ice for 30 min in Radio Immunoprecipitation Assay (RIPA) lysis buffer, followed by centrifugation at 12,000 *g* for 20 min at 4°C. Supernatants were collected, and total protein was evidenced using a bicinchoninic acid (BCA) assay. Equal amounts of total protein (15 μg) were mixed with protein loading buffer, denatured at 100°C for 15 min, separated by Sodium Dodecyl Sulfate PolyAcrylamide Gel Electrophoresis (SDS-PAGE), and then transferred to polyvinylidene fluoride (PVDF) membranes. Membranes were blocked using 5% non-fat milk for 2 h at room temperature, incubated overnight at 4°C with primary antibody, washed with Tris-Borate-Sodium Tween-20 (TBST), and then incubated with secondary antibody for 1 h at room temperature, followed by another wash with TBST (Xiao et al., 2023). Proteins were detected by an electrochemiluminescence detection system. The images were analyzed with ImageJ 1.8 software (National Institutes of Health, Bethesda, MD, USA; https://imagej.nih.gov/ij).

### 2.7 Plaque assay

MDCK cells were grown to confluence and infected with diluted cell culture supernatants for 2 h. The supernatants were then aspirated, and an agarose overlay was applied, containing 1X DMEM, 1% low-melting-point agarose (Sigma–Aldrich, St. Louis, MO, USA, Cat# [A6877-25G]), and 2 μg/mL TPCK-treated trypsin. After 72 h of incubation at 37°C, cells were fixed with 4% paraformaldehyde for 1 h and then stained with crystal violet (Wang et al., 2024).

### 2.8 Fluorescence microscopy

Following 2 h of infection, the inoculum was removed, and cells were cultured for 24 h in F12 maintenance medium containing either OTS167 or DMSO. Cells were then washed 3 times with sterile PBS (5 min each wash). Cells were fixed with 4% paraformaldehyde for 10 min, washed 3 times with sterile PBS (5 min each wash), and permeabilized with 1% Triton X-100 in PBS for 4 min. After three washes with sterile PBS (5 min each wash), cells were blocked with 1% BSA in PBS at room temperature for 1 h and washed 3 times with sterile PBS (5 min each wash). Cells were then incubated overnight at 4°C with primary antibody (NP, 1:300), washed 3 times with sterile PBS (5 min each wash), and incubated for 1 h at room temperature with secondary fluorescent antibody (1:100). After three washes with sterile PBS (5 min each wash), nuclei were counterstained with 4′, 6-diamidino-2-phenylindole (DAPI) for 5 min, and washed 3 times with sterile PBS (Cui et al., 2024). Images of cells were captured using an inverted fluorescence microscope (Zeiss Axio Vert.A1, Shanghai, China). Two filters were used, the green fluorescent protein (GFP) channel for whole-virus visualization and the DAPI channel for nuclear visualization.

### 2.9 Small interfering RNA study

A549 cells were plated in 6-well plates and cultured to near 80% confluence. Cells were transfected with siRNA targeting MELK (siRNA1: 5′-CCUAGUACUGCAAUUCGGGAAAUUU-3′; siRNA2: 5′-CCCAAAUGGAAACCAGGAA-3′) using Lipofectamine 2000 Transfection Reagent (Invitrogen, Shanghai, China), following the manufacturer’s protocol. Non-targeting siRNA served as a negative control (El-Mayet et al., 2024). Western blot assay was conducted as previously described to confirm gene knockdown at 12 h post-transfection.

### 2.10 *In vivo* anti-influenza activity of OTS167

Seven-week-old female C57BL/6 mice were divided into three groups, and 10^3^ TCID50 PR/8 viruses were intranasally inoculated into each mouse at a volume of 50 μL. Subsequently, OTS167 suspended in H_2_O was intranasally administered to mice at 10 mg/kg concentrations for 3 consecutive days (Yang et al., 2023). Body weight and survival of the infected mice were monitored following infection.

### 2.11 Synergy analysis

The combined effects of oseltamivir, zanamivir, and OTS167 on IAV PR/8 replication were evaluated using the Bliss independence model in SynergyFinder 3.0 (Institute for Molecular Medicine Finland, Helsinki, Finland; https://synergyfinder.fimm.fi) (Ianevski et al., 2022). Data were obtained from A549 cells infected with IAV PR/8 and treated with drug combinations for 24 h. A synergy score between −10 and 10 was considered additive, a score greater than 10 suggested synergism, and a score less than −10 indicated antagonism. A 95% confidence interval was used to determine statistical significance.

### 2.12 Statistical analysis

Quantitative results are reported as mean ± standard error of the mean (SEM). Intergroup comparisons were performed using the Mann–Whitney U test in GraphPad Prism 8. Significance is marked in the figures using asterisks: ^*^p < 0.05, ^**^p < 0.01, and ^***^p < 0.001.

## 3 Results

### 3.1 Screening kinase inhibitors identified the MELK inhibitor OTS167 against IAV infection

**Figure 1:**
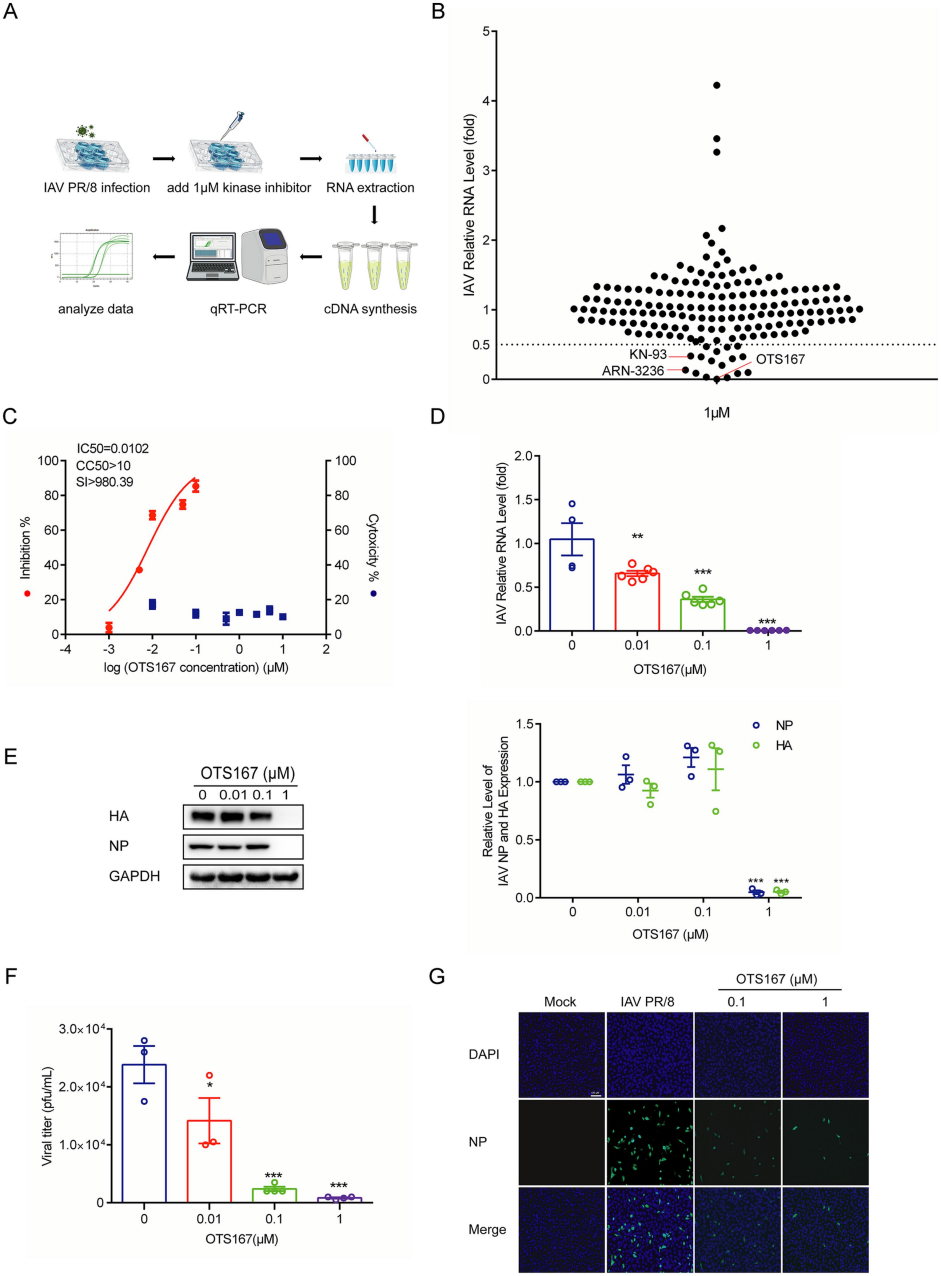
Screening a library of kinase inhibitors identified OTS167 as a potent inhibitor against influenza A PR/8 virus infection. **(A,B)** A549 cells were infected with the PR/8 virus. Following infection, the cells were treated with 172 kinase inhibitors at a concentration of 1 μM for 36 h. RT-qPCR analysis of viral RNA revealed that OTS167 exhibited significant inhibitory activity against PR/8 virus replication. **(C)** CC_50_ curves of OTS167 were determined by CCK-8 assays on A549 cells. IC_50_ of OTS167 was determined through plaque assay. **(D)** Dose-dependent inhibitory activity of OTS167 on PR/8 virus-infected A549 cells. RT-qPCR data were normalized to the reference gene GAPDH and presented relative to the control. **(E)** Influenza PR/8 virus-infected A549 cells were treated with different concentrations of OTS167, and protein samples were harvested after 36 h. The expression of viral NP and HA was stained and evidenced by western blot assay. **(F)** OTS167 dose-dependent inhibition of viral titer. **(G)** Indirect fluorescence microscope analysis of viral NP (green) upon treatment with OTS167. Nuclei were visualized by DAPI (blue). Data are presented as the means ± SEM (*n* = 3, ^*^p < 0.05, ^**^p < 0.01, and ^***^p < 0.001).

To probe the role of human kinome in influenza virus infection, we screened a library of 172 pharmacological inhibitors targeting a large number of protein kinases. We inoculated the human A549 lung cell line with the IAV PR/8 strain. To minimize non-specific effects on host cells, we used a relatively low concentration of 1 μM and treated for 36 h. By RT-qPCR quantification of viral genomic RNA, we identified 17 candidates exerting over 50% inhibitory effects, and 10 with inhibition over 70%. Among these, OTS167 was one of the most potent candidates (Figures 1A,B). Subsequently, we determined the half maximal inhibitory concentration (IC_50_) against IAV PR/8 as 0.0102 μM, and the half maximal cytotoxic concentration (CC_50_) of OTS167 in A549 cells as over 10 μM. This corresponds to a selectivity index (SI) of over 980, indicating a huge therapeutic window (Figure 1C). Treatment of IAV PR/8-infected A549 cells with 0.01, 0.1, and 1 μM OTS167 significantly inhibited viral replication in a dose-dependent manner (Figure 1D). OTS167 markedly suppressed the synthesis of NP and HA proteins (Figure 1E). Plaque assay revealed that OTS167 effectively inhibited the production of infectious viral particles in the cell culture supernatant (Figure 1F). For example, treatment with 1 μM for 24 h reduced the infectious viral titer by over 90% (Figure 1F).

To further investigate the effects of OTS167 on the PR/8 virus, OTS167 was administered at three distinct time points (Supplementary Figure S1A). The expression of viral HA and NP proteins was evaluated 24 h after either OTS167 pre-treatment 2 h before IAV infection, or during co-incubation of OTS167 with IAV (Supplementary Figure S1B). Additionally, the expression of viral HA and NP proteins was assessed at 6, 8, and 10 h post-infection when cells were treated with OTS167 after IAV infection (Supplementary Figure S1C). Results suggest that OTS167 may exert its regulatory effects and impact viral replication immediately following its addition. Indirect immunofluorescence analysis of NP protein expression (green fluorescence) showed reduced fluorescence at both 0.1 and 1 μM concentrations of OTS167 treatment (Figure 1G). These findings suggest that, although most kinase inhibitors exhibit limited antiviral activity, MELK represents a promising target, and its pharmacological inhibitor OTS167 exerts potent anti-influenza activity.

### 3.2 Influenza viral replication activates MELK to support the infection

**Figure 2:**
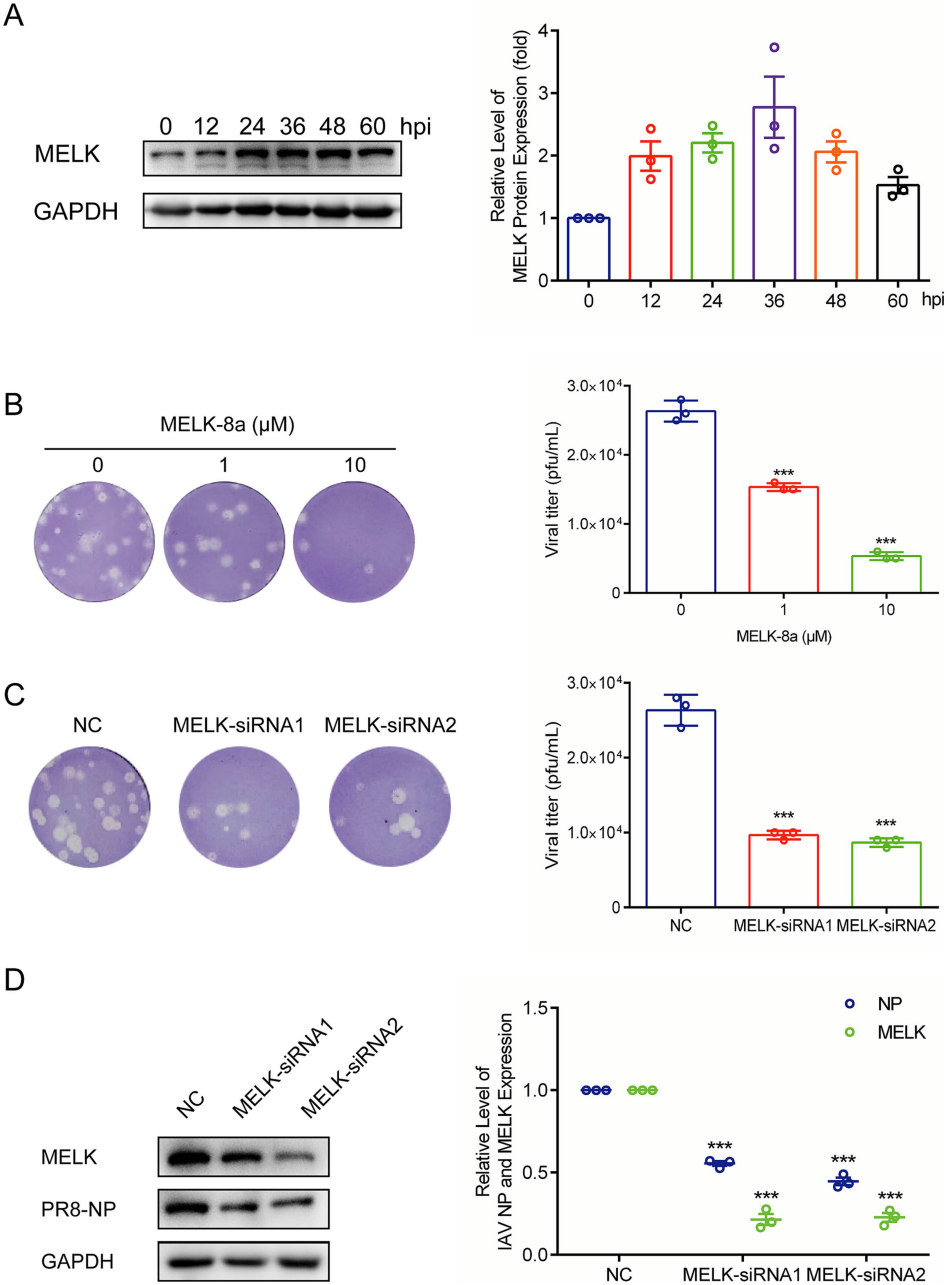
MELK is activated during influenza virus replication. **(A)** IAV PR/8 infected A549 cells, and protein samples were harvested after 0, 12, 24, 36, 48, and 60 h. The expression of MELK protein was stained and evidenced by western blot assay. **(B)** MELK-8a, another MELK inhibitor, was employed to treat IAV-infected cells at doses of 1 and 10 μM. Cell culture supernatant was analyzed by plaque assay. **(C,D)** Following transfection with 200 pmol siRNA-MELK and negative control (NC), protein samples and cell supernatants were collected 24 h post-infection with IAV-PR/8. Western blot was performed to assess the expression of MELK and NP proteins, and plaque assay was conducted to evaluate the production of viral particles. Data are presented as the means ± SEM (*n* = 3, ^*^p < 0.05, ^**^p < 0.01, and ^***^p < 0.001).

To probe whether influenza regulates MELK expression, we evidenced MELK protein levels at a series of time points after IAV PR/8 inoculation in A549 cells. We observed that MELK protein expression was upregulated over time during the replication of IAV PR/8 (Figure 2A). Furthermore, treatment with OTS167 profoundly inhibited MELK expression in A549 cells infected with IAV PR/8 (Supplementary Figures S2A,B). To further validate the anti-influenza potential of targeting MELK, we employed another pharmacological inhibitor, MELK-8a. Consistently, plaque assay showed the inhibitory effects on influenza virus replication at concentrations of 1 and 10 μM (Figure 2B).

To confirm MELK’s role, we performed siRNA-mediated gene silencing in A549 cells. As shown in Figure 2D, MELK expression was significantly reduced in knockdown cells compared to that in the scramble control cells. Importantly, MELK knockdown significantly impaired IAV PR/8 replication as shown by reduced viral NP protein levels and effectively inhibited the production of infectious viral particles into cell culture supernatant at 12 h post-infection (Figures 2C,D). Viral titers in supernatants were evidenced by plaque assay. These results indicate that MELK is a host factor supporting influenza virus replication, which can be targeted by the pharmacological inhibitors, including OTS167 and MELK-8a.

### 3.3 The MELK inhibitor OTS167 regulates CDK1 to interfere with viral mRNA splicing

**Figure 3:**
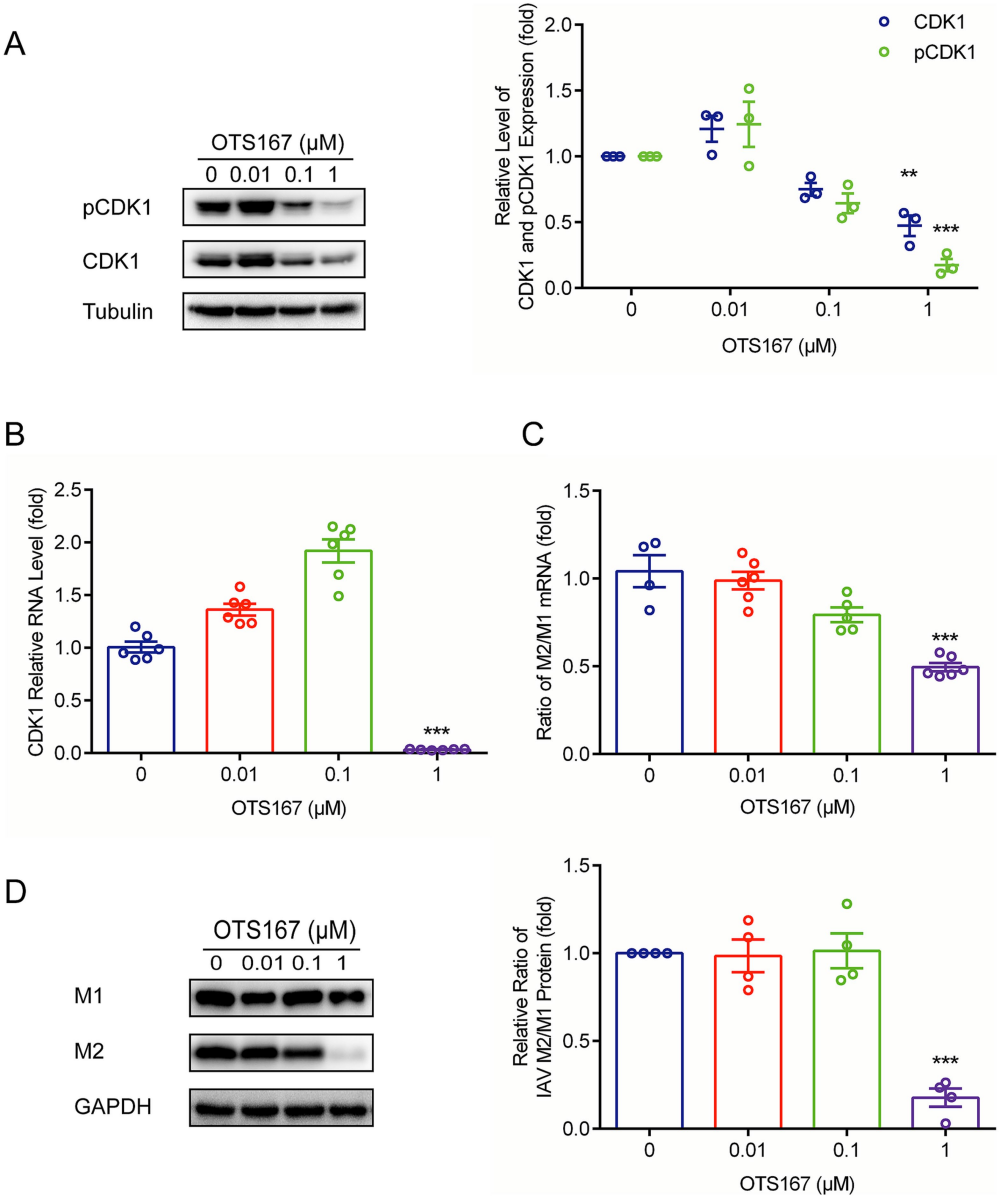
OTS167 suppresses M1 mRNA splicing by downregulating CDK1. **(A)** CDK1 accumulation was inhibited by OTS167. Lysates of IAV-infected A549 cells were subjected to western blot for CDK1 and pCDK1. **(B)** OTS167 significantly reduced CDK1 mRNA levels. RT-qPCR data were normalized to the reference gene GAPDH. **(C)** OTS167 blocks M1 splicing. IAV-PR8 M2 and M1 mRNA were evidenced by RT-qPCR after 24 h of OTS167 treatment. **(D)** M2 and M1 protein levels were detected by western blot, and the expression levels were analyzed. Data are presented as the means ± SEM (*n* = 3, ^*^p < 0.05, ^**^p < 0.01, and ^***^p < 0.001).

It has been reported that MELK regulates CDK1 (also known as cdc2 kinase) (Maes et al., 2019). Cdc2-like kinase 1 (CLK1) has been recognized as a potential anti-influenza target owing to alternative splicing of the M1 gene in influenza viruses (Karlas et al., 2010; Li et al., 2018). CDK1 is reported to regulate the function of spliceosomes in cells by phosphorylating the splicing factor SF2/ASF (Okamoto et al., 1998). Therefore, we further investigated the impact of OTS167 on CDK1 and its phosphorylated form. We found that both CDK1 and phosphorylated CDK1 protein levels were significantly inhibited by OTS167 treatment (Figure 3A). Reduction in pCDK1 levels correlated with total CDK1 downregulation, implying MELK inhibition destabilizes CDK1 protein. The precise mechanisms underlying the reduction of both total CDK1 and its phosphorylated forms remain to be further elucidated. Furthermore, the observed upregulation of CDK1 transcription levels at low OTS167 concentrations may stem from protein functional deficiency, triggering cellular compensatory feedback mechanisms, potentially through enhanced RNA synthesis to counteract functional impairment (Figure 3B).

We then examined the splicing of influenza M1 mRNA. As shown in Figure 3C, the ratio of M2/M1 mRNA was reduced by OTS167, and consequently, the accumulation of M2 protein declined, resulting in a decreased ratio of M2/M1 protein expression (Figure 3D). This finding indicated that the antiviral activity of OTS167 against the influenza virus is at least partially through affecting the splicing of M1 viral RNAs.

### 3.4 Broad-spectrum antiviral activity of OTS167 against both IAV and IBV strains

**Figure 4:**
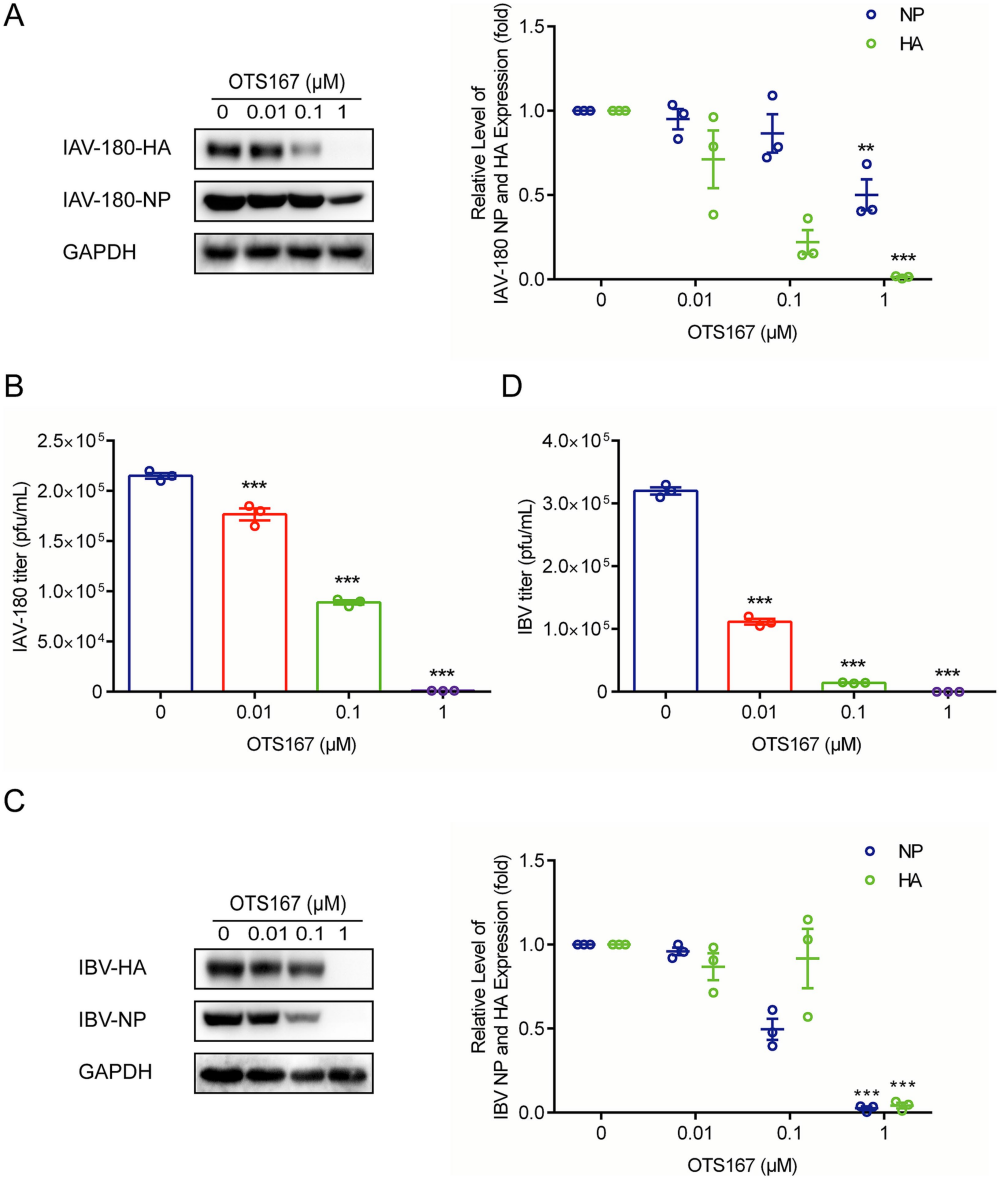
Antiviral activity of OTS167 against various IAV and IBV strains. **(A)** IAV-180-infected A549 cells were treated with different concentrations of OTS167, and protein samples were harvested after 24 h. The expression of viral NP and HA was stained and evidenced by Western blot assay. **(B)** OTS167 significantly reduced IAV-180 virion production. Cell culture supernatant was analyzed by plaque assay. **(C)** IBV-Washington-infected A549 cells were treated with different concentrations of OTS167, and protein samples were harvested after 24 h. The expression of viral NP and HA was stained and evidenced by Western blot assay. **(D)** OTS167 significantly reduced IBV-Washington virion production. Cell culture supernatant was analyzed by plaque assay. Data are presented as the means ± SEM (*n* = 3, ^*^p < 0.05, ^**^p < 0.01, and ^***^p < 0.001).

To further investigate whether targeting MELK by OTS167 has broad anti-influenza virus activity, we further evaluated its effects on another IAV strain, IAV-180, and an IBV strain, B/Washington/02/2019. Western blotting of viral protein and plaque assays, and quantification of infectious titers showed consistent antiviral activity. At a concentration of 1 μM, the expression of IAV-180 HA and NP proteins was inhibited by more than 90 and 50%, respectively, while the expression of IBV HA and NP proteins was inhibited by more than 90% in each case (Figures 4A,C). Simultaneously, the production of both IAV-180 and IBV viral particles was inhibited by over 90% (Figures 4B,D). These findings suggest that targeting MELK by OTS167 exerts broad-spectrum antiviral effects against multiple influenza virus strains of IAV and IBV.

### 3.5 OTS167 exhibits anti-influenza virus activity *in vivo*

**Figure 5:**
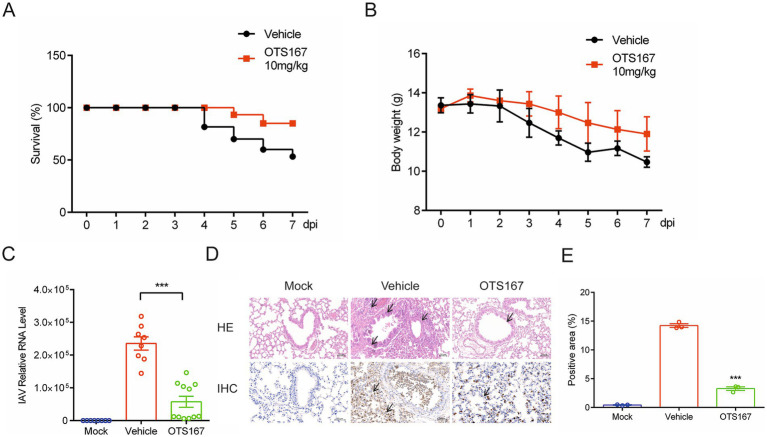
*In vivo* antiviral effects of OTS167 against IAV. **(A)** Survival curves. Mice were infected with PR/8 in the presence of OTS167 at indicated concentrations or H_2_O. **(B)** Weight curves. Throughout the experimental period, mouse weight was recorded daily for a maximum of 7 days. **(C)** RT-qPCR was used to measure the viral load in the lungs. RT-qPCR data were normalized to the reference gene GAPDH and presented relative to the control. **(D)** H&E staining and immunohistochemistry assay of sectioned lungs. At 7 days post-infection, pathological changes were evaluated based on neutrophil infiltration and necrosis using H&E staining. The viral antigens were visualized using NP-antibodies via an immunohistochemistry assay. **(E)** The PR8 NP protein was analyzed using ImageJ software for visualization purposes. Data are presented as the means ± SEM (*n* = 3, ^*^p < 0.05, ^**^p < 0.01, and ^***^p < 0.001).

Subsequently, we evaluated the *in vivo* anti-influenza effects of OTS167 in a mouse model. Briefly, C57BL/6 mice were infected with the influenza A PR/8 strain via intranasal administration, and they were treated once daily with OTS167 or vehicle. Notably, treatment with OTS167 at 10 mg/kg for 3 days delayed virus-induced weight loss and reduced mortality (Figures 5A,B). OTS167 treatment significantly reduced viral loads by approximately 70% compared to vehicle (Figure 5C). Histological examination of lung tissues obtained from necropsied mice 7 days post-infection (dpi) revealed neutrophil infiltration and necrosis with inflammatory cell infiltration. As expected, treatment with OTS167 decreased infection-induced pathological lesions in the lungs. We further performed an immunohistochemistry assay using specific anti-IAV antibodies to visualize the viruses in stained images. As shown in Figure 5D, relatively few viral antigens were detected in the OTS167 treatment group compared to those in the vehicle group. Compared to the infection group, the NP protein-positive area was significantly reduced in OTS167-treated mice (Figure 5E). No toxic effects were observed in the lungs, liver, or kidneys following the administration of OTS167 alone (Supplementary Figure S3). Thus, treatment with OTS167 inhibited influenza virus infection and prolonged the survival of infected mice.

### 3.6 Combinatory effects of OTS167 and anti-flu drug on IAV replication

The clinic often uses combination antiviral therapies to achieve optimal antiviral efficacy and avoid resistance development. Because of their distinct antiviral mechanisms, we tested the combination of OTS167 with the clinically used anti-flu drugs oseltamivir and zanamivir.

**Figure 6:**
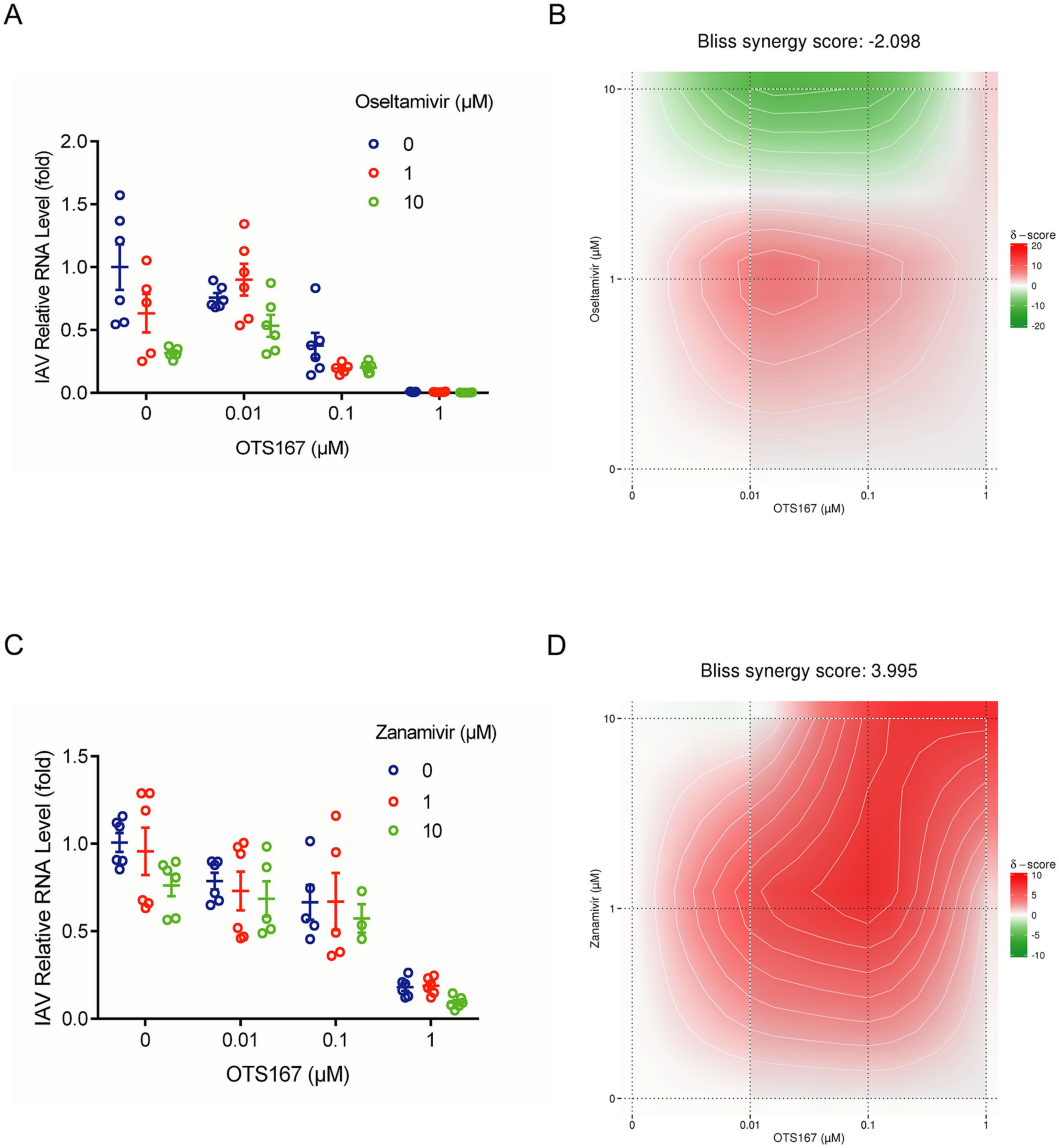
Combinatory effects of OTS167 and anti-flu drug on IAV replication. **(A,B)** After OTS167 was used in combination with oseltamivir concentration, the viral mRNA level was detected by RT-qPCR. **(C,D)** Through the analysis of the combination drug data, the score was −2.098, showing additive antiviral activity. After OTS167 was used in combination with zanamivir concentration, the viral mRNA level was detected by RT-qPCR. Through the analysis of the combination drug data, the score was 3.995, showing additive antiviral activity. *N* = 3 independent experiments with two or three replicates each.

We first determined the non-toxic concentrations of oseltamivir and zanamivir (Supplementary Figure S4). Subsequently, we assessed the combined inhibitory effects of OTS167 with oseltamivir and zanamivir against IAV PR/8 using RT-qPCR to measure IAV relative RNA levels (Figures 6A,C). The Bliss synergy score was calculated as −2.098 (Figure 6B), indicating additive antiviral activity against IAV by combining OTS167 and oseltamivir. The Bliss synergy score was calculated as 3.995 (Figure 6D), indicating additive antiviral activity against IAV by combining OTS167 and zanamivir. Thus, these two selected anti-flu agents showed additive antiviral effects in combination with OTS167.

## 4 Discussion

These existing therapies for treating influenza predominantly consist of direct-acting antivirals (DAAs) that target specific viral proteins, including NA, PA, and M2. Given the rise of drug-resistant variants, it is imperative to identify new anti-influenza strategies and potential therapeutics. The host-targeted broad-spectrum antiviral strategy represents an attractive potential solution to overcome these limitations. Host kinase inhibitors represent one category of compounds with great potential to be repurposed as broad-spectrum antivirals. Viruses hijack a large number of host kinases at distinct steps of their life cycle (Supekova et al., 2008; Li et al., 2009; Keating and Striker, 2012; Jiang et al., 2014). Due to the broad requirement of some host kinases, they represent promising targets for broad-spectrum therapies. The development and approval of a substantial number of kinase inhibitors for cancer (Gross et al., 2015) and inflammatory conditions (Ott and Adams, 2011), in conjunction with these findings, has driven efforts to assess the therapeutic potential of such compounds against viral infections.

**Figure 7:**
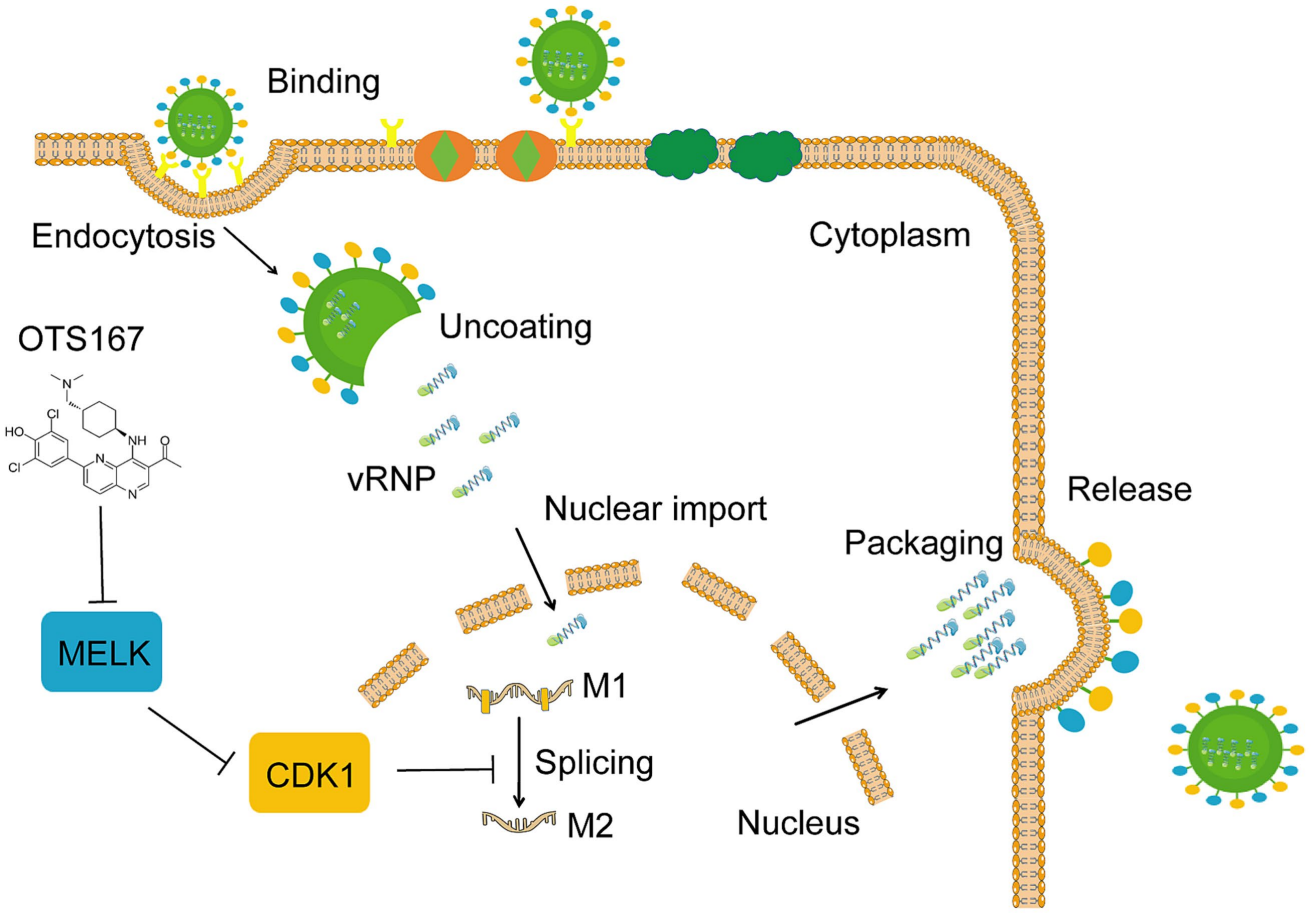
Schematic representation of the proposed cascade of OTS167 inhibition of IAV replication. OTS167 inhibits MELK, regulating to the downstream effector CDK1, thereby inhibiting influenza virus M1 mRNA splicing to reduce viral replication and particle assembly.

This study utilized A549 cells infected with IAV PR/8 as a model to evaluate the impact of kinase inhibitors on IAV infection. Following the screening of 172 kinase inhibitors, we found that most of these inhibitors did not exhibit significant inhibition of viral replication. Notably, OTS167, a MELK inhibitor, demonstrated a pronounced antiviral effect against multiple strains of influenza viruses. The mechanistic investigation revealed that OTS167 inhibits influenza virus infection by affecting CDK1, thus suppressing the splicing of the M1 gene (Figure 7).

MELK phosphorylates MELK itself, and this autophosphorylation contributes to MELK stability. OTS167 has been described as inhibiting MELK by blocking autophosphorylation of MELK, thus resulting in the degradation and loss of MELK protein (Chung et al., 2016). We observed MELK protein degradation and loss following OTS167 treatment (Supplementary Figure S2A). To validate MELK as a critical node in influenza virus replication, we employed another MELK inhibitor, MELK-8a (Touré et al., 2016). Treatment with MELK-8a demonstrated dose-dependent inhibition of progeny virus production (Figure 2B). Concurrently, we inhibited the expression of MELK in A549 cells via siRNA-mediated knockdown. The analysis showed that MELK siRNA significantly attenuated viral protein expression and progeny virion production (Figures 2C,D).

Previous studies have demonstrated that MELK downstream regulates CDK1, a kinase involved in viral M1 mRNA splicing (Maes et al., 2019; Zhao et al., 2022). We found that both CDK1 and phosphorylated CDK1 protein levels were significantly inhibited by OTS167 treatment (Figure 3A). Subsequently, we measured the yields of M2/M1 mRNA and the corresponding protein and found that OTS167 can disrupt the M1 mRNA splicing for impairing IAV infection (Figures 3C,D). These results support that OTS167 exhibits substantial antiviral activity, making it a potential candidate for antiviral drug development. This effect may be attributed to the inhibition of MELK and subsequent modulation of M1 mRNA splicing.

MELK regulates splicing events through a mitotic phosphorylation-dependent interaction with the transcription and splicing factor NIPP1, a protein phosphatase 1 inhibitor (Vulsteke et al., 2004). Currently, MELK has been predominantly studied in the context of tumorigenesis and cancer therapy (Su et al., 2024). Additionally, previous studies have reported that MELK is involved in the uncoating process of HIV, regulating the replication of nucleic acids during the later stages of the viral lifecycle, and plays a crucial role in the HIV replication cycle (Takeuchi et al., 2017; Nishiyama et al., 2022). This suggests a potential analogous mechanism of action for OTS167 against the influenza virus, a possibility that warrants further investigation. Concurrently, the “cytokine storm” associated with severe influenza represents a primary factor contributing to increased mortality rates. Therefore, it is imperative to consider not only the inhibition of viral replication but also the mitigation of pulmonary inflammatory responses in the clinical treatment of influenza. Protein kinases also play significant roles in inflammation-related signaling pathways, regulating the host’s immune responses to pathogen stimuli to activate pathways that combat viruses and suppress inflammation (Arthur and Ley, 2013; Zarrin et al., 2021). In this study, we found that OTS167 can alleviate the level of inflammation in the lungs of mice (Figure 5D). Nuclear factor kappa-B (NF-κB) regulates various aspects of innate and adaptive immune functions and is a critical mediator of the inflammatory response (Barnabei et al., 2021). MELK can modulate the NF-κB pathway through SQSTM1 (Janostiak et al., 2017), suggesting a potential link between these studies. Furthermore, MELK can also regulate C-Jun (Ganguly et al., 2015; Maes et al., 2019), which is associated with influenza virus replication and host inflammation (Xie et al., 2014).

The combination therapy approach is also an essential antiviral strategy in clinical settings. Resistance to antiviral agents frequently occurs during influenza treatment; however, combining medications may yield improved therapeutic outcomes (Nguyen et al., 2010; O’Hanlon et al., 2019). Compared to monotherapy, we found additive antiviral activity of OTS167 in combination with both zanamivir and oseltamivir (Figures 6B,D). These findings may inform the development and clinical application of novel antiviral therapeutics.

In summary, screening of a kinase inhibitor library identified OTS167 as a potent inhibitor of influenza virus infection. Mechanistically, inhibiting MELK by OTS167 downregulates the expression of CDK1 to interfere with viral M1 mRNA splicing. However, the exact mechanisms by which MELK regulates influenza virus infection require further investigation. Given that OTS167 is already under clinical investigation for treating cancer, our results support the repurposing of OTS167 for influenza treatment, particularly in immunocompromised cancer patients who are at high risk of acquiring infection.

## Data Availability

The original contributions presented in the study are included in the article/Supplementary material, further inquiries can be directed to the corresponding author.

## References

[ref1] Arthur J. S. C. Ley S. C. (2013). Mitogen-activated protein kinases in innate immunity. Nat. Rev. Immunol. 13, 679–692. doi: 10.1038/nri3495, PMID: 23954936

[ref2] Barnabei L. Laplantine E. Mbongo W. Rieux-Laucat F. Weil R. (2021). NF-κB: at the borders of autoimmunity and inflammation. Front. Immunol. 12:716469. doi: 10.3389/fimmu.2021.716469, PMID: 34434197 PMC8381650

[ref3] Chen S. Wang Y. Li P. Yin Y. Bijvelds M. J. de Jonge H. R.. (2020). Drug screening identifies gemcitabine inhibiting rotavirus through alteration of pyrimidine nucleotide synthesis pathway. Antivir. Res. 180:104823. doi: 10.1016/j.antiviral.2020.104823, PMID: 32485209 PMC7261112

[ref4] Chung S. Kijima K. Kudo A. Fujisawa Y. Harada Y. Taira A.. (2016). Preclinical evaluation of biomarkers associated with antitumor activity of MELK inhibitor. Oncotarget 7, 18171–18182. doi: 10.18632/oncotarget.7685, PMID: 26918358 PMC4951280

[ref5] Cui Z. Zhang J. Wang J. Liu J. Sun P. Li J.. (2024). Caffeic acid phenethyl ester: an effective antiviral agent against porcine reproductive and respiratory syndrome virus. Antivir. Res. 225:105868. doi: 10.1016/j.antiviral.2024.105868, PMID: 38490343

[ref6] Dey S. Mondal A. (2024). Unveiling the role of host kinases at different steps of influenza A virus life cycle. J. Virol. 98:e0119223. doi: 10.1128/jvi.01192-23, PMID: 38174932 PMC10805039

[ref7] Dubois J. Terrier O. Rosa-Calatrava M. (2014). Influenza viruses and mRNA splicing: doing more with less. mBio 5:e00070-14. doi: 10.1128/mBio.00070-14, PMID: 24825008 PMC4030477

[ref8] Eisenberg E. Levanon E. Y. (2013). Human housekeeping genes, revisited. Trends Genet. 29, 569–574. doi: 10.1016/j.tig.2013.05.010, PMID: 23810203

[ref9] el-Mayet F. Santos V. C. Wijesekera N. Lubbers S. Harrison K. S. Sadeghi H.. (2024). Glucocorticoid receptor and specificity protein 1 (Sp1) or Sp3, but not the antibiotic mithramycin A, stimulates human alphaherpesvirus 1 (HSV-1) replication. Antivir. Res. 225:105870. doi: 10.1016/j.antiviral.2024.105870, PMID: 38556059 PMC11109923

[ref10] Ganguly R. Mohyeldin A. Thiel J. Kornblum H. I. Beullens M. Nakano I. (2015). MELK—a conserved kinase: functions, signaling, cancer, and controversy. Clin. Transl. Med. 4:11. doi: 10.1186/s40169-014-0045-y, PMID: 25852826 PMC4385133

[ref11] Garg S. Reinhart K. Couture A. Kniss K. Davis C. T. Kirby M. K.. (2024). Highly pathogenic avian influenza A(H5N1) virus infections in humans. N. Engl. J. Med. 392, 843–854. doi: 10.1056/NEJMoa2414610 39740051

[ref12] Gross S. Rahal R. Stransky N. Lengauer C. Hoeflich K. P. (2015). Targeting cancer with kinase inhibitors. J. Clin. Invest. 125, 1780–1789. doi: 10.1172/JCI76094, PMID: 25932675 PMC4463189

[ref13] Hui X. Cao L. Xu T. Zhao L. Huang K. Zou Z.. (2022). PSMD12-mediated M1 ubiquitination of influenza A virus at K102 regulates viral replication. J. Virol. 96:e0078622. doi: 10.1128/jvi.00786-22, PMID: 35861516 PMC9364790

[ref14] Ianevski A. Giri A. K. Aittokallio T. (2022). Synergy finder 3.0: an interactive analysis and consensus interpretation of multi-drug synergies across multiple samples. Nucleic Acids Res. 50, W739–W743. doi: 10.1093/nar/gkac382, PMID: 35580060 PMC9252834

[ref15] Janostiak R. Rauniyar N. Lam T. T. Ou J. Zhu L. J. Green M. R.. (2017). MELK promotes melanoma growth by stimulating the NF-κB pathway. Cell Rep. 21, 2829–2841. doi: 10.1016/j.celrep.2017.11.033, PMID: 29212029 PMC5726781

[ref16] Jassem A. N. Roberts A. Tyson J. Zlosnik J. E. A. Russell S. L. Caleta J. M.. (2024). Critical illness in an adolescent with influenza A(H5N1) virus infection. N. Engl. J. Med. 392, 927–929. doi: 10.1056/NEJMc2415890 39740022

[ref17] Javanian M. Barary M. Ghebrehewet S. Koppolu V. Vasigala V. Ebrahimpour S. (2021). A brief review of influenza virus infection. J. Med. Virol. 93, 4638–4646. doi: 10.1002/jmv.26990, PMID: 33792930

[ref18] Jiang W.-M. Zhang X.-Y. Zhang Y.-Z. Liu L. Lu H.-Z. (2014). A high throughput RNAi screen reveals determinants of HIV-1 activity in host kinases. Int. J. Clin. Exp. Pathol. 7, 2229–2237. 24966931 PMC4069921

[ref19] Karlas A. Machuy N. Shin Y. Pleissner K.-P. Artarini A. Heuer D.. (2010). Genome-wide RNAi screen identifies human host factors crucial for influenza virus replication. Nature 463, 818–822. doi: 10.1038/nature08760, PMID: 20081832

[ref20] Keating J. A. Striker R. (2012). Phosphorylation events during viral infections provide potential therapeutic targets. Rev. Med. Virol. 22, 166–181. doi: 10.1002/rmv.722, PMID: 22113983 PMC3334462

[ref21] Kupferschmidt K. (2024). Why hasn’t the bird flu pandemic started? Science 386, 1205–1206. doi: 10.1126/science.adv2422, PMID: 39666804

[ref22] Li Q. Brass A. L. Ng A. Hu Z. Xavier R. J. Liang T. J.. (2009). A genome-wide genetic screen for host factors required for hepatitis C virus propagation. Proc. Natl. Acad. Sci. U.S.A. 106, 16410–16415. doi: 10.1073/pnas.0907439106, PMID: 19717417 PMC2752535

[ref23] Li C.-C. Wang X.-J. Wang H.-C. R. (2019). Repurposing host-based therapeutics to control coronavirus and influenza virus. Drug Discov. Today 24, 726–736. doi: 10.1016/j.drudis.2019.01.018, PMID: 30711575 PMC7108273

[ref24] Li C. Xu L.-J. Lian W.-W. Pang X.-C. Jia H. Liu A.-L.. (2018). Anti-influenza effect and action mechanisms of the chemical constituent gallocatechin-7-gallate from *Pithecellobium clypearia* Benth. Acta Pharmacol. Sin. 39, 1913–1922. doi: 10.1038/s41401-018-0030-x, PMID: 29802302 PMC6289332

[ref25] Liang Y. (2023). Pathogenicity and virulence of influenza. Virulence 14:2223057. doi: 10.1080/21505594.2023.2223057, PMID: 37339323 PMC10283447

[ref26] Livak K. J. Schmittgen T. D. (2001). Analysis of relative gene expression data using real-time quantitative PCR and the 2(−Delta Delta C(T)) method. Methods 25, 402–408. doi: 10.1006/meth.2001.1262, PMID: 11846609

[ref27] Maes A. Maes K. Vlummens P. De Raeve H. Devin J. Szablewski V.. (2019). Maternal embryonic leucine zipper kinase is a novel target for diffuse large B cell lymphoma and mantle cell lymphoma. Blood Cancer J. 9:87. doi: 10.1038/s41408-019-0249-x, PMID: 31740676 PMC6861269

[ref28] Meineke R. Rimmelzwaan G. F. Elbahesh H. (2019). Influenza virus infections and cellular kinases. Viruses 11:171. doi: 10.3390/v11020171, PMID: 30791550 PMC6410056

[ref29] Nguyen J. T. Hoopes J. D. Le M. H. Smee D. F. Patick A. K. Faix D. J.. (2010). Triple combination of amantadine, ribavirin, and oseltamivir is highly active and synergistic against drug resistant influenza virus strains in vitro. PLoS One 5:e9332. doi: 10.1371/journal.pone.0009332 20179772 PMC2825274

[ref30] Nishiyama T. Takada T. Takeuchi H. Iwami S. (2022). Maternal embryonic leucine zipper kinase (MELK) optimally regulates the HIV-1 uncoating process. J. Theor. Biol. 545:111152. doi: 10.1016/j.jtbi.2022.111152, PMID: 35545145

[ref31] O’Hanlon R. Leyva-Grado V. H. Sourisseau M. Evans M. J. Shaw M. L. (2019). An influenza virus entry inhibitor targets class II PI3 kinase and synergizes with oseltamivir. ACS Infect. Dis. 5, 1779–1793. doi: 10.1021/acsinfecdis.9b00230, PMID: 31448902

[ref32] Okamoto Y. Onogi H. Honda R. Yasuda H. Wakabayashi T. Nimura Y.. (1998). cdc2 kinase-mediated phosphorylation of splicing factor SF2/ASF. Biochem. Biophys. Res. Commun. 249, 872–878. doi: 10.1006/bbrc.1998.9247, PMID: 9731229

[ref33] Ott P. A. Adams S. (2011). Small-molecule protein kinase inhibitors and their effects on the immune system: implications for cancer treatment. Immunotherapy 3, 213–227. doi: 10.2217/imt.10.99, PMID: 21322760 PMC4009988

[ref34] Roskoski R. (2024). Properties of FDA-approved small molecule protein kinase inhibitors: a 2024 update. Pharmacol. Res. 200:107059. doi: 10.1016/j.phrs.2024.107059, PMID: 38216005

[ref35] Su H.-C. Feng I.-J. Tang H.-J. Shih M.-F. Hua Y.-M. (2022). Comparative effectiveness of neuraminidase inhibitors in patients with influenza: a systematic review and network meta-analysis. J. Infect. Chemother. 28, 158–169. doi: 10.1016/j.jiac.2021.11.014, PMID: 34840038

[ref36] Su P. Lu Q. Wang Y. Mou Y. Jin W. (2024). Targeting MELK in tumor cells and tumor microenvironment: from function and mechanism to therapeutic application. Clin. Transl. Oncol. 27, 887–900. doi: 10.1007/s12094-024-03664-5, PMID: 39187643

[ref37] Sueca-Comes M. Rusu E. C. Grabowska A. M. Bates D. O. (2022). Looking under the lamppost: the search for new cancer targets in the human kinome. Pharmacol. Rev. 74, 1136–1145. doi: 10.1124/pharmrev.121.000410, PMID: 36180110

[ref38] Supekova L. Supek F. Lee J. Chen S. Gray N. Pezacki J. P.. (2008). Identification of human kinases involved in hepatitis C virus replication by small interference RNA library screening. J. Biol. Chem. 283, 29–36. doi: 10.1074/jbc.M703988200, PMID: 17951261

[ref39] Takeuchi H. Saito H. Noda T. Miyamoto T. Yoshinaga T. Terahara K.. (2017). Phosphorylation of the HIV-1 capsid by MELK triggers uncoating to promote viral cDNA synthesis. PLoS Pathog. 13:e1006441. doi: 10.1371/journal.ppat.1006441, PMID: 28683086 PMC5500366

[ref40] Touré B. B. Giraldes J. Smith T. Sprague E. R. Wang Y. Mathieu S.. (2016). Toward the validation of maternal embryonic leucine zipper kinase: discovery, optimization of highly potent and selective inhibitors, and preliminary biology insight. J. Med. Chem. 59, 4711–4723. doi: 10.1021/acs.jmedchem.6b00052, PMID: 27187609

[ref41] Tsai P.-L. Chiou N.-T. Kuss S. García-Sastre A. Lynch K. W. Fontoura B. M. A. (2013). Cellular RNA binding proteins NS1-BP and hnRNP K regulate influenza A virus RNA splicing. PLoS Pathog. 9:e1003460. doi: 10.1371/journal.ppat.1003460, PMID: 23825951 PMC3694860

[ref42] Uyeki T. M. Milton S. Abdul Hamid C. Reinoso Webb C. Presley S. M. Shetty V.. (2024). Highly pathogenic avian influenza A(H5N1) virus infection in a dairy farm worker. N. Engl. J. Med. 390, 2028–2029. doi: 10.1056/NEJMc2405371, PMID: 38700506

[ref43] Vulsteke V. Beullens M. Boudrez A. Keppens S. Van Eynde A. Rider M. H.. (2004). Inhibition of spliceosome assembly by the cell cycle-regulated protein kinase MELK and involvement of splicing factor NIPP1. J. Biol. Chem. 279, 8642–8647. doi: 10.1074/jbc.M311466200, PMID: 14699119

[ref44] Wang X. Pu F. Yang X. Feng X. Zhang J. Duan K.. (2024). Immunosuppressants exert antiviral effects against influenza A(H1N1)pdm09 virus via inhibition of nucleic acid synthesis, mRNA splicing, and protein stability. Virulence 15:2301242. doi: 10.1080/21505594.2023.2301242, PMID: 38170681 PMC10854267

[ref45] Xiao Y. Yan Y. Chang L. Ji H. Sun H. Song S.. (2023). CDK4/6 inhibitor palbociclib promotes SARS-CoV-2 cell entry by down-regulating SKP2 dependent ACE2 degradation. Antivir. Res. 212:105558. doi: 10.1016/j.antiviral.2023.105558, PMID: 36806814 PMC9938000

[ref46] Xie J. Zhang S. Hu Y. Li D. Cui J. Xue J.. (2014). Regulatory roles of c-jun in H5N1 influenza virus replication and host inflammation. Biochim. Biophys. Acta 1842, 2479–2488. doi: 10.1016/j.bbadis.2014.04.017, PMID: 24780373

[ref47] Yang X. Long F. Jia W. Zhang M. Su G. Liao M.. (2023). Artesunate inhibits PDE4 leading to intracellular cAMP accumulation, reduced ERK/MAPK signaling, and blockade of influenza A virus vRNP nuclear export. Antivir. Res. 215:105635. doi: 10.1016/j.antiviral.2023.105635, PMID: 37192683

[ref48] Zarrin A. A. Bao K. Lupardus P. Vucic D. (2021). Kinase inhibition in autoimmunity and inflammation. Nat. Rev. Drug Discov. 20, 39–63. doi: 10.1038/s41573-020-0082-8, PMID: 33077936 PMC7569567

[ref49] Zhao L. Yan Y. Dai Q. Wang Z. Yin J. Xu Y.. (2022). The CDK1 inhibitor, Ro-3306, is a potential antiviral candidate against influenza virus infection. Antivir. Res. 201:105296. doi: 10.1016/j.antiviral.2022.105296, PMID: 35367281

