# Supplementary material for: Screening kinase inhibitors identifies MELK as a prime target against influenza virus infections through inhibition of viral mRNA splicing
Source: Front Microbiol. 2025 Jun 5;16:1600935. doi: 10.3389/fmicb.2025.1600935 (PMC12176825; doi:10.3389/fmicb.2025.1600935)
Supplement: Supplementary file 1 [file Image_1.pdf]

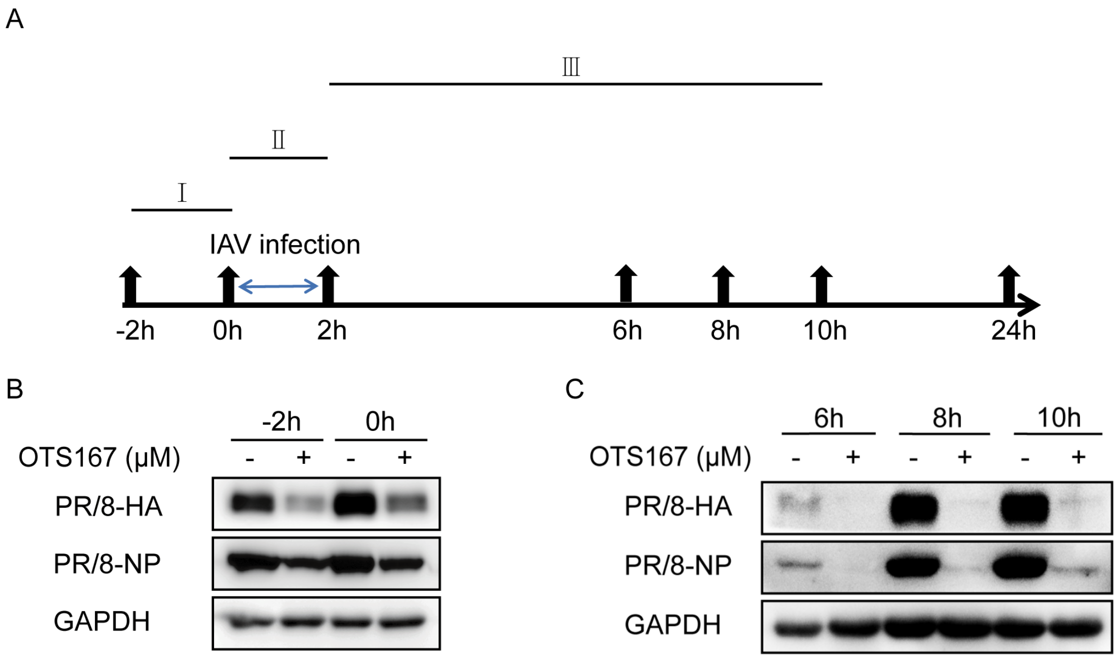

Figure S1 OTS167 reduces viral HA and NP protein expression at various time points. (A) OTS167 was administered at three distinct time points. All samples were infected with the influenza PR/8 strain between 0 hour and 2 hours, and the course of OTS167 addition was set into five intervals (Ⅰ–Ⅲ). (B) OTS167 was administered 2 hours prior to or co-incubated with IAV PR/8, and Western blot detects HA and NP proteins 24 hours after viral infection. (C) At 6, 8, and 10 hours following treatment with OTS167, both HA and NP protein levels were found to be reduced.

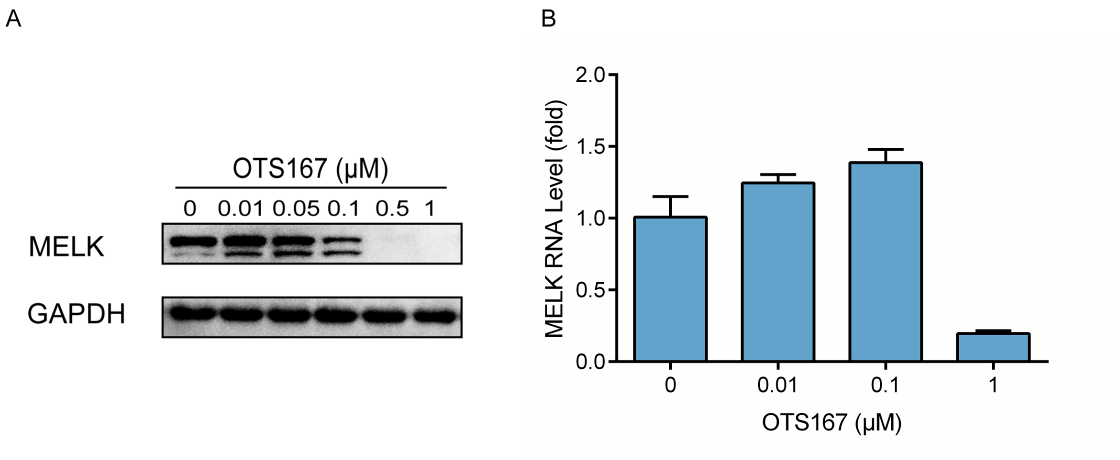

Figure S2 OTS167 significantly inhibits MELK. (A) Western blot analysis showed that OTS167 substantially reduces MELK protein expression. (B) qRT-PCR analysis revealed that 1 μM OTS167 significantly decreases MELK mRNA level.

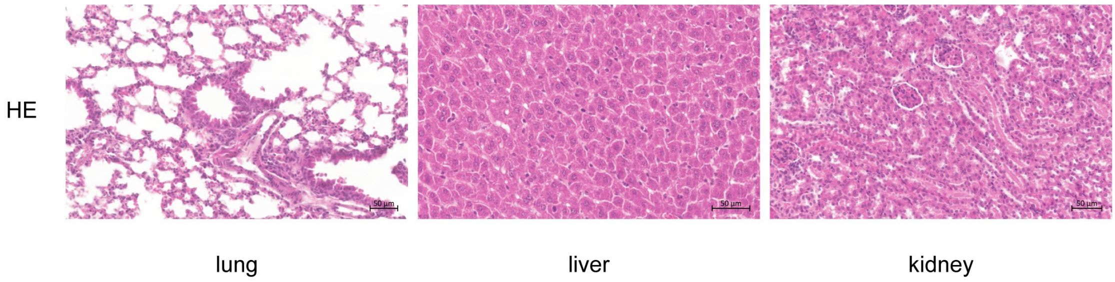

Figure S3 The toxic effect of OTS167 on mice. The first group was given OTS167 (10mg/kg/d) for 3 days. All the mice were euthanized on 7 dpi. Their lung, liver and kidney tissues were isolated and examined using histopathology (scale bar 50 μm).

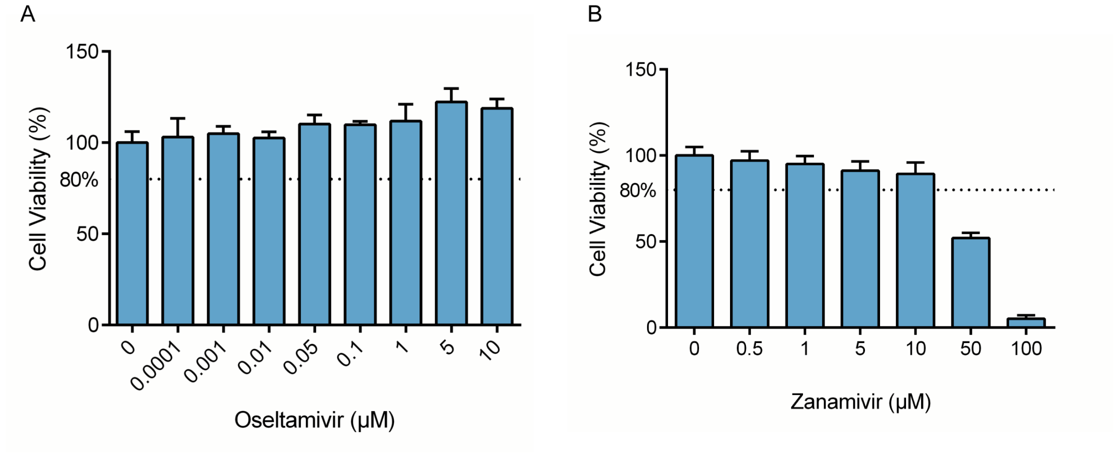

Figure S4 The cytotoxicity of oseltamivir and zanamivir on A549 cells. A549 cells treated with different concentrations of oseltamivir and zanamivir for 24 hours. Cell viability was determined by CCK-8 assay.
