# Supplementary material for: Screening kinase inhibitors identifies MELK as a prime target against influenza virus infections through inhibition of viral mRNA splicing
Source: Front Microbiol. 2025 Jun 5;16:1600935. doi: 10.3389/fmicb.2025.1600935 (PMC12176825; doi:10.3389/fmicb.2025.1600935)
Supplement: Supplementary file 2 [file Table_1.DOCX]

**Supplementary table 1.** **List of kinase inhibitors (KIs) library.**

|  | Name | Kinase Target | Relative RNA Level |
| --- | --- | --- | --- |
| 1 | AIM-100 | Ack1 | 0.5872 |
| 2 | GNF-7 |  | 0.4000 |
| 3 | 5-Iodotubercidin | Adenosine Kinase | 0.8516 |
| 4 | ABT-702 dihydrochloride |  | 1.2956 |
| 5 | BAY1125976 | Akt | 0.7994 |
| 6 | M2698 |  | 1.4720 |
| 7 | BAY-3827 | AMPK | 0.7390 |
| 8 | MRT199665 |  | 3.4570 |
| 9 | Repotrectinib | Anaplastic lymphoma kinase | 1.6021 |
| 10 | NVP-TAE 684 |  | 1.0616 |
| 11 | AZD0156 | ATM/ATR | 0.9402 |
| 12 | Elimusertib |  | 0.3253 |
| 13 | PF 477736 | Aurora Kinase | 1.0307 |
| 14 | Barasertib |  | 0.9592 |
| 15 | BV02 | Bcr-Abl | 1.0273 |
| 16 | Asciminib |  | 0.7549 |
| 17 | CHMFL-BMX-078 | BMX Kinase | 1.2113 |
| 18 | BMX-IN-1 |  | 0.7358 |
| 19 | PCI-33380 | Btk | 1.7142 |
| 20 | Dihydrodiol-Ibrutinib |  | 1.2116 |
| 21 | Cabamiquine | CAMK | 1.5266 |
| 22 | KN-93 |  | 0.3353 |
| 23 | TBCA | Casein Kinase | 0.6086 |
| 24 | SR-3029 |  | 0.0243 |
| 25 | Simurosertib | CDK | 0.6457 |
| 26 | Fadraciclib |  | 0.0327 |
| 27 | PLX5622 | c-Fms | 0.9009 |
| 28 | Edicotinib |  | 0.6317 |
| 29 | GDC-0575 | Checkpoint Kinase (Chk) | 1.9579 |
| 30 | AZD-7762 |  | 0.6547 |
| 31 | Avapritinib | c-Kit | 0.7179 |
| 32 | Motesanib |  | 0.4626 |
| 33 | JNJ-38877618 | c-Met/HGFR | 1.1907 |
| 34 | Glumetinib |  | 1.1089 |
| 35 | TNIK-IN-3 | DAPK | 1.4916 |
| 36 | HS38 |  | 0.9679 |
| 37 | BAY-826 | Discoidin Domain Receptor | 1.2330 |
| 38 | WRG-28 |  | 0.8730 |
| 39 | Samotolisib | DNA-PK | 0.9321 |
| 40 | CC-115 |  | 0.6434 |
| 41 | Harmine (hydrochloride) | DYRK  DYRK | 1.0344 |
| 42 | Harmine |  | 0.5872 |
| 43 | AEE788 | EGFR | 1.6369 |
| 44 | Rezivertinib |  | 1.3981 |
| 45 | JI-101 | Ephrin Receptor | 0.8064 |
| 46 | NVP-BHG712 |  | 0.2651 |
| 47 | Tauroursodeoxycholate | ERK | 1.5713 |
| 48 | Lidocaine |  | 1.0828 |
| 49 | Narmafotinib | FAK | 1.8300 |
| 50 | Chloropyramine hydrochloride |  | 0.8246 |
| 51 | Infigratinib | FGFR | 0.8800 |
| 52 | Sulfatinib |  | 0.8194 |
| 53 | Fostamatinib | FLT3 | 0.8885 |
| 54 | Gilteritinib |  | 0.6612 |
| 55 | PF-04937319 | Glucokinase | 1.3092 |
| 56 | IHVR-11029 |  | 1.0327 |
| 57 | Tideglusib | GSK-3 | 1.0641 |
| 58 | Cromolyn (sodium) |  | 0.9665 |
| 59 | LDN-209929 (dihydrochloride) | Haspin Kinase | 0.8607 |
| 60 | CHR-6494 |  | 0.0847 |
| 61 | Lonidamine | Hexokinase | 1.4033 |
| 62 | 3-Bromopyruvic acid |  | 1.2388 |
| 63 | AX-024 (hydrochloride) | IFNAR | 0.9406 |
| 64 | IFN alpha-IFNAR-IN-1 (hydrochloride) |  | 0.6958 |
| 65 | Linsitinib | IGF-1R | 0.7344 |
| 66 | BMS-754807 |  | 0.4776 |
| 67 | Tizoxanide | IKK | 0.5736 |
| 68 | Vinpocetine |  | 0.3227 |
| 69 | AGL-2263 | Insulin Receptor | 0.6178 |
| 70 | MID-1 |  | 0.1999 |
| 71 | Emavusertib | IRAK | 1.2829 |
| 72 | IRAK4-IN-7 |  | 0.9886 |
| 73 | Kira8 | IRE1 | 0.9517 |
| 74 | Sunitinib |  | 0.8806 |
| 75 | PF-06465469 | Itk | 1.0996 |
| 76 | BMS-509744 |  | 1.0061 |
| 77 | Abrocitinib | JAK | 1.7583 |
| 78 | Fedratinib |  | 0.6517 |
| 79 | Tanzisertib | JNK | 1.3229 |
| 80 | Bentamapimod |  | 0.8051 |
| 81 | BMS-5 | LIM Kinase (LIMK) | 0.8076 |
| 82 | T56-LIMKi |  | 0.7687 |
| 83 | PFE-360 | LRRK2 | 1.3827 |
| 84 | GSK2578215A |  | 1.0127 |
| 85 | Selonsertib | MAP3K | 1.1120 |
| 86 | Takinib |  | 0.9657 |
| 87 | PF-3644022 | MAPKAPK2 (MK2) | 1.2225 |
| 88 | CMPD1 |  | 0.4713 |
| 89 | Trametinib | MEK | 1.5821 |
| 90 | Cobimetinib |  | 1.3901 |
| 91 | JNJ-47117096 hydrochloride | MELK | 1.2432 |
| 92 | OTS167 |  | 0.0038 |
| 93 | TC13172 | Mixed Lineage Kinase | 1.2666 |
| 94 | Necrosulfonamide |  | 1.1835 |
| 95 | Tomivosertib | MNK | 1.1545 |
| 96 | Tinodasertib |  | 1.1122 |
| 97 | Mps-BAY2a | Mps1 | 1.2933 |
| 98 | Empesertib |  | 0.8721 |
| 99 | Temsirolimus | mTOR | 1.1446 |
| 100 | Pamapimod | p38 MAPK | 1.2973 |
| 101 | Talmapimod |  | 0.7366 |
| 102 | PF-3758309 | PAK | 1.2489 |
| 103 | 5-Aminosalicylic Acid |  | 1.0264 |
| 104 | Axitinib | PDGFR | 1.3382 |
| 105 | Trapidil |  | 0.5588 |
| 106 | JX06 | PDHK | 0.9141 |
| 107 | AZD7545 |  | 0.8181 |
| 108 | BX-912 | PDK-1 | 3.2645 |
| 109 | MP7 |  | 1.3335 |
| 110 | GSK2606414 | PERK | 0.9955 |
| 111 | Opnurasib |  | 0.9367 |
| 112 | Alpelisib | PI3K | 1.0579 |
| 113 | Idelalisib |  | 0.7623 |
| 114 | BQR-695 | PI4K | 1.5316 |
| 115 | MMV390048 |  | 0.8867 |
| 116 | Apilimod | PIKfyve | 0.8443 |
| 117 | Vacuolin-1 |  | 0.2970 |
| 118 | Uzansertib (phosphate) | Pim | 1.2003 |
| 119 | TP-3654 |  | 0.6427 |
| 120 | Fasudil (Hydrochloride) | PKA | 1.1292 |
| 121 | Staurosporine |  | 0.0883 |
| 122 | Midostaurin | PKC | 1.4873 |
| 123 | Ruboxistaurin (hydrochloride) |  | 0.8525 |
| 124 | CID755673 | PKD | 1.5005 |
| 125 | CID 2011756 |  | 0.7565 |
| 126 | Volasertib | Polo-like Kinase (PLK) | 1.6471 |
| 127 | Onvansertib |  | 0.7195 |
| 128 | PF-4618433 | Pyk2 | 0.8338 |
| 129 | Shikonin | Pyruvate Kinase | 4.2259 |
| 130 | PKM2-IN-1 |  | 0.8507 |
| 131 | Regorafenib | Raf | 1.3153 |
| 132 | Sorafenib |  | 1.1600 |
| 133 | Sotorasib | Ras | 0.9774 |
| 134 | Lonafarnib |  | 0.9531 |
| 135 | Pralsetinib | RET | 2.0679 |
| 136 | Selpercatinib |  | 1.0278 |
| 137 | LY-2584702 (tosylate salt) | Ribosomal S6 Kinase (RSK) | 1.0504 |
| 138 | Sodium Salicylate |  | 0.7380 |
| 139 | GSK2982772 | RIP kinase | 1.3303 |
| 140 | GSK3145095 |  | 0.7859 |
| 141 | Belumosudil | ROCK | 0.9693 |
| 142 | Ripasudil |  | 0.6201 |
| 143 | GGTI-2418 | ROS Kinase | 0.9983 |
| 144 | Taletrectinib |  | 0.5428 |
| 145 | YKL-05-099 | Salt-inducible Kinase (SIK) | 1.0454 |
| 146 | ARN-3236 |  | 0.1332 |
| 147 | GSK 650394 | SGK | 1.2889 |
| 148 | EMD638683 |  | 1.1527 |
| 149 | Peretinoin | SPHK | 1.4827 |
| 150 | PF-543 |  | 1.3035 |
| 151 | Tirbanibulin | Src | 1.4163 |
| 152 | Saracatinib |  | 1.1228 |
| 153 | MSC-1186 | SRPK | 1.3495 |
| 154 | SPHINX |  | 1.0409 |
| 155 | Fludarabine | STAT | 1.1635 |
| 156 | Pimozide |  | 0.6328 |
| 157 | Entospletinib | Syk | 2.1673 |
| 158 | Sovleplenib |  | 1.3937 |
| 159 | Tamnorzatinib | TAM Receptor | 0.9511 |
| 160 | Dubermatinib |  | 0.0979 |
| 161 | Galunisertib | TGF-β Receptor | 1.3990 |
| 162 | Vactosertib |  | 1.0107 |
| 163 | Ilaprazole (sodium) | TOPK | 1.0459 |
| 164 | Cephradine |  | 0.8121 |
| 165 | Larotrectinib | Trk Receptor | 1.4632 |
| 166 | Selitrectinib |  | 0.6808 |
| 167 | ULK-101 | ULK | 1.2412 |
| 168 | GW406108X |  | 0.9662 |
| 169 | Vandetanib | VEGFR | 1.2368 |
| 170 | Tivozanib |  | 1.0968 |
| 171 | RP-6306 | Wee1 | 0.9803 |
| 172 | Adavosertib |  | 0.9413 |

Supplementary table 2. qRT-PCR primers used in the study, from 5' to 3'.

| Gene name | Sense primer | Antisense primer |
| --- | --- | --- |
| IAV PR/8-PB1 | CATCACTGGAGATAACAC | GGAGCAATACTTAGAACAT |
| IAV PR/8-M1 | ATGCCCTTAATGGGAACGGG | ATGAGGCCCATACAACTGGC |
| IAV PR/8-M2 | GAAAGGAGGGCCTTCTACGG | GTCAGCATCCACAGCACTCT |
| MELK | AATCGTTACACTACACCCTCA | CTCTCAGGGCTAATGACACC |
| Human GAPDH | CTCTGGTAAAGTGGATATTGT | GGTGGAATCATATTGGAACA |
